# Safety and immunogenicity of neoadjuvant treatment using WT1-immunotherapeutic in combination with standard therapy in patients with WT1-positive Stage II/III breast cancer: a randomized Phase I study

**DOI:** 10.1007/s10549-017-4130-y

**Published:** 2017-02-07

**Authors:** M. Higgins, G. Curigliano, V. Dieras, S. Kuemmel, G. Kunz, P. A. Fasching, M. Campone, T. Bachelot, P. Krivorotko, S. Chan, A. Ferro, L. Schwartzberg, M. Gillet, P. M. De Sousa Alves, V. Wascotte, F. F. Lehmann, P. Goss

**Affiliations:** 1 Department of Medical Oncology, Mater Misericordiae University Hospital, Eccles St., Dublin, 7 Ireland; 2 Division of Early Drug Development, Istituto Europeo di Oncologia, Milan, Italy; 3 Clinical Investigational Unit, Institut Curie, Paris, France; 4 Breast UnitKliniken Essen-Mitte, Essen, Germany; 5 Department of Obstetrics & Gynecology, St.-Johannes-Hospital, Dortmund, Germany; 6 Department of Gynecology and Obstetrics, University Hospital Erlangen, Comprehensive Cancer Center Erlangen-EMN, Friedrich-Alexander University Erlangen-EMN, Erlangen, Germany; 7 Service d’Oncologie Médicale, Institut de Cancérologie de l’Ouest, Saint Herblain, France; 8 Department of Medical Oncology, Centre Leon Berard, Lyon, France; 9 Petrov Research Institute of Oncology, St. Petersburg, Russian Federation; 10 Department of Clinical Oncology, Nottingham University Hospital NHS Trust (City campus), Nottingham, UK; 11 Unit of Medical Oncology, Santa Chiara Hospital, Trento, Italy; 12 Division of Hematology/Oncology, University of Tennessee Health Science Center, The West Clinic, Medical Oncology, Memphis, USA; 13 GSK Vaccines, Rixensart, Belgium; 14 Celyad, Mont-Saint-Guibert, Belgium; 15 MGH Cancer Center, Massachusetts General Hospital, Boston, USA

**Keywords:** Breast cancer, Immunotherapy, Neoadjuvant therapy, WT1 antigen, Immunogenicity, Safety

## Abstract

**Purpose:**

This Phase I, multicenter, randomized study (ClinicalTrials.gov NCT01220128) evaluated the safety and immunogenicity of recombinant Wilms’ tumor 1 (WT1) protein combined with the immunostimulant AS15 (WT1-immunotherapeutic) as neoadjuvant therapy administered concurrently with standard treatments in WT1-positive breast cancer patients.

**Methods:**

Patients were treated in 4 cohorts according to neoadjuvant treatment (A: post-menopausal, hormone receptor [HR]-positive patients receiving aromatase inhibitors; B: patients receiving chemotherapy; C: HER2-overexpressing patients on trastuzumab–chemotherapy combination; D: HR-positive/HER2-negative patients on chemotherapy). Patients (cohorts A–C) were randomized (2:1) to receive 6 or 8 doses of WT1-immunotherapeutic or placebo together with standard neoadjuvant treatment in a double-blind manner; cohort D patients received WT1-immunotherapeutic in an open manner. Safety was assessed throughout the study. WT1-specific antibodies were assessed pre- and post-vaccination.

**Results:**

Sixty-two patients were randomized; 60 received ≥ one dose of WT1-immunotherapeutic. Two severe toxicities were reported: diarrhea (cohort C; also reported as a grade 3 serious adverse event) and decreased left ventricular ejection fraction (cohort B; also reported as a grade 2 adverse event). Post-dose 4 of WT1-immunotherapeutic, 10/10 patients from cohort A, 0/8 patients from cohort B, 6/11 patients from cohort C, and 2/3 patients from cohort D were humoral responders. The sponsor elected to close the trial prematurely.

**Conclusions:**

Concurrent administration of WT1-immunotherapeutic and standard neoadjuvant therapy was well tolerated and induced WT1-specific antibodies in patients receiving neoadjuvant aromatase inhibitors. In patients on neoadjuvant chemotherapy or trastuzumab–chemotherapy combination, the humoral response was impaired or blunted, likely due to either co-administration of corticosteroids and/or the chemotherapies themselves.

**Electronic supplementary material:**

The online version of this article (doi:10.1007/s10549-017-4130-y) contains supplementary material, which is available to authorized users.

## Introduction

Immunotherapies are rapidly becoming standard of care for many solid tumors. In the last 5 years, ipilimumab, pembrolizumab, and nivolumab have been approved for many cancer types [1–4]. There is an evolving interest in the immunogenicity of breast tumors and the possible role of immunotherapy in this common cancer [5, 6]. Various immunotherapeutic strategies, including checkpoint inhibitors, vaccines, adoptive T-cell transfer, or cytokine therapy, have been tested for treatment of breast cancer (BC) [6, 7]. Vaccines constitute an attractive immunotherapy approach aiming to stimulate the intrinsic antitumor immune response by presenting tumor antigens recognized by T-cells. Wilms’ tumor 1 (WT1) is a potential target antigen for cancer immunotherapy as it is over-expressed in the majority of solid tumors [8–12]. Owing to its specificity, oncogenicity, immunogenicity, and therapeutic function, WT1 has been classified as one of the most promising targets for cancer immunotherapy [13]. WT1 plays an oncogenic role in BC and is expressed in approximately 33% (range: 3–48.5%) of malignant breast tumors [11, 14–16]. Additionally, high WT1 levels have previously been correlated with poorer outcomes in BC [15, 17].

Combining chemotherapy with immune-based interventions has great potential for optimizing clinical outcomes of BC patients. This study evaluated the safety, immunogenicity, and preliminary clinical activity of the WT1 antigen combined with GSK’s proprietary immunostimulant AS15 (WT1-immunotherapeutic) administered to women with BC during standard neoadjuvant treatment.

## Patients and methods

### Study design and patients

This study was an international, multicenter, double-blind, randomized, placebo-controlled, Phase I/II clinical trial conducted between 2011 and 2014 in 19 medical centers in Belgium, France, Germany, Italy, the Russian federation, the United Kingdom, and the United States. Phase I initially included three parallel cohorts (A, B, and C), in which patients were randomized in a double-blind manner (2:1) to receive six or eight doses of WT1-immunotherapeutic (WT1 groups) or placebo (placebo groups) at 3-week intervals, together with the standard neoadjuvant treatment (Fig. S1).

The neoadjuvant treatment was chosen according to institutional standards, based on the hormone receptor (HR) and human epidermal growth factor receptor-2 (HER2) status of the tumor. Cohort A received daily aromatase inhibitors (AIs) for 18 or 24 weeks of neoadjuvant treatment; cohorts B and C received WT1-immunotherapeutic/placebo on the same day as chemotherapy (Fig. S2), with patients in cohort C also receiving trastuzumab. Further recruitment beyond Phase I in each cohort depended on the outcome of intermediate assessment of the induced WT1-specific antibody response. Only if a ≥40% response rate (based on post-dose 4 WT1-specific antibody responses in at least six patients in the WT1 group) was achieved, and provided no safety issues were identified, would the cohort proceed to Phase II.

Following the analysis of early immunogenicity results in cohort B (see Results section), a further cohort (D) was opened to investigate an alternative dosing schedule (Fig. S1). Cohort D received WT1-immunotherapeutic on day 14 of each 3-weekly chemotherapy cycle in an open-label manner (Fig. S2).

Patients aged ≥18 years with WT1-positive, histologically confirmed, primary invasive BC were eligible for enrollment. Details of inclusion/exclusion criteria, as well as study treatment and administration, study procedures, data collection, and blood sampling are included in Supplementary materials.

All patients provided written informed consent before any study-related procedures. The study was conducted in accordance with Good Clinical Practice and all applicable regulatory requirements, including the Declaration of Helsinki. The protocol was approved by the national independent ethics committees and institutional review boards of the study centers. The study was registered at www.ClinicalTrials.gov (NCT01220128). A protocol summary is available at http://www.gsk-clinicalstudyregister.com (GSK study ID 113172).

### Objectives

Phase I study objectives were the evaluation of safety and immunogenicity of WT1-immunotherapeutic as neoadjuvant therapy administered concurrently with different standard treatments.

Phase II objectives included further assessment of safety and immunogenicity, and a preliminary assessment of the clinical activity of WT1-immunotherapeutic in combination with standard neoadjuvant treatments, i.e., pathological complete response (pCR) rate, disease free survival (DFS), and overall survival (OS); of note, due to early termination of the trial, the analysis of DFS and OS outcomes was not performed.

### Safety and immunogenicity assessments

Adverse events (AEs), including severe toxicities (defined in Supplementary materials), and serious adverse events (SAEs) were assessed throughout the study.

WT1-specific antibodies were measured by an enzyme-linked immunosorbent assay (ELISA). WT1-specific humoral response was defined as the appearance of antibodies for baseline seronegative patients, or an at least 2-fold increase in antibody concentrations for baseline seropositive patients. The ELISA assay cut-off was 9 ELISA units (EU)/ml.

### Clinical activity assessment

pCR, i.e., complete response (CR) or partial response (PR) in the breast and axillary nodes was assessed at the definitive surgery. pCR in the primary tumor was evaluated according to the Miller/Payne grading system [18], and in lymph nodes, by histopathological examination. The reference pCR rates based on the reported in literature rates under standard treatment for a given patient population were: 5% for cohort A (based on a 3–5% rate), 20% for cohort B (6–30%), and 50% for cohort C (30–65%) (see details in Supplementary materials).

### Statistical analyses

Statistical analyses were performed using Statistical Analysis Systems (SAS) Drug and Development with SAS version 9.2.

The total treated cohort (TTC) included all patients who received at least one dose of WT1-immunotherapeutic/placebo. The according-to-protocol (ATP) cohort for immunogenicity included all eligible patients (i.e., those meeting all eligibility criteria for enrollment), who did not report major protocol deviation, who received at least the first four doses of WT1-immunotherapeutic/placebo, and who provided a valid result for immunogenicity measurement within four weeks of post-dose 4 (visit 5). Data collected after major protocol violation were eliminated from ATP immunogenicity analyses.

Descriptive analyses of demographics and baseline characteristics were performed on the TTC. Safety analyses were performed on the TTC, and immunogenicity analyses on the ATP cohort for immunogenicity.

## Results

### Study patients

Phase I recruitment was completed in March 2013 for cohort A, November 2011 for cohort B, and June 2012 for cohort C. Phase II recruitment for cohort A had been initiated as the protocol criteria were met, but was stopped prematurely in July 2014, following the sponsor’s decision. Enrollment in cohort B did not proceed to the Phase II segment because the protocol-defined immune response success (≥40% of patients showing a humoral response) was not fulfilled. In cohort C, weak immune responses with antibody concentrations close to the assay cut-off values were induced in only a few patients (see *Immunogenicity* section below) and, although meeting the protocol criteria of success, these immune responses were considered sub-optimal; therefore, Phase II for this cohort was not initiated. Recruitment of cohort D patients was also stopped prematurely at the same time as the Phase II for cohort A.

**Fig. 1:**
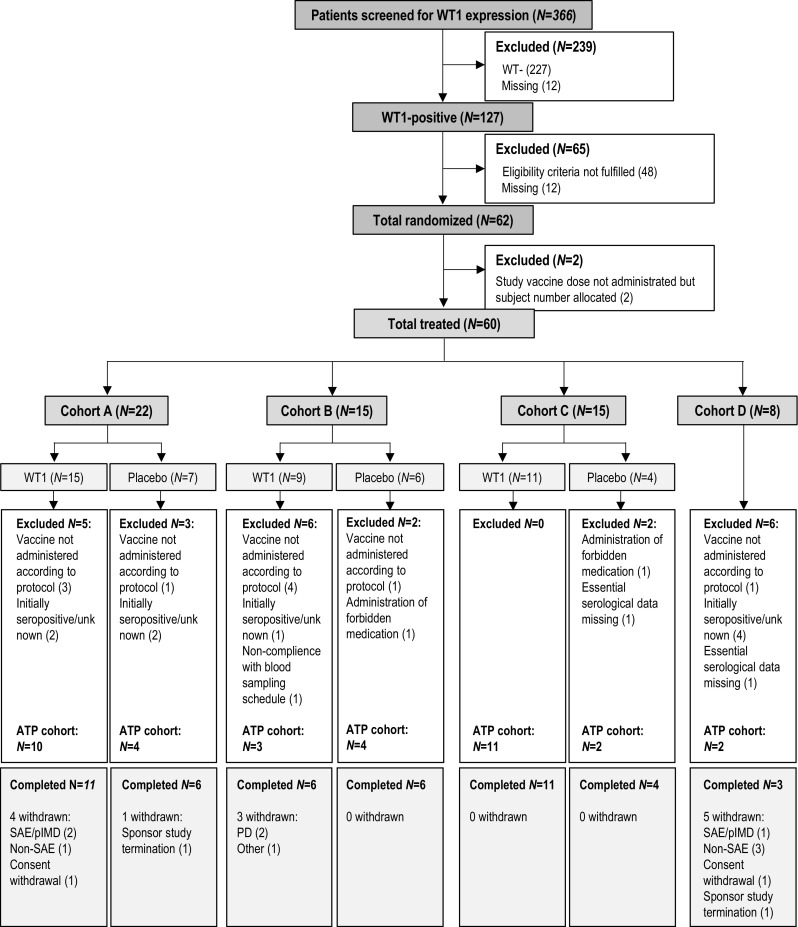
Participant flow *N*, number of patients; *WT1* patients who received WT1-immunotherapeutic; *ATP* cohort, according-to-protocol cohort for immunogenicity; *SAE* serious adverse event; *pIMD* potential immune-mediated disease; *PD* progressive disease; Cohort A: post-menopausal patients with hormone receptor-positive breast cancer receiving AIs as neoadjuvant therapy; Cohort B: patients receiving neoadjuvant chemotherapy; Cohort C: patients with human epidermal growth factor receptor-2 (HER-2)-overexpressing breast cancer receiving neoadjuvant trastuzumab therapy combined with chemotherapy; Cohort D: patients with hormone receptor-positive/HER2-negative breast cancer receiving neoadjuvant chemotherapy; patients in cohort D received WT1-immunotherapeutic in an open-label manner

In total, 366 patients were screened for WT1 expression; 127 (34.7%) had WT1-positive tumors. Sixty-two patients were randomized and 60 were treated (cohort A: 22, B: 15, C: 15, D: 8); 47 patients completed the treatment (Fig. 1).

The majority of patients (95.0%) were of Caucasian origin; the median age (range) of the patients in WT1 and placebo groups was 72.0 (54–84) and 74.0 (60–80) years in cohort A, 41.0 (37–77) and 62.5 (48–74) years in cohort B, 52 (38–69) and 53.0 (46–61) years in cohort C, respectively, and 47 (42–69) years in cohort D (WT1 group only). The majority of patients enrolled had Stage IIA (38.3%) or IIB (38.3) tumors; 13.3% had Stage IIIA, 8.3%, Stage IIIB, and 1.7%, Stage IIIC tumors.

### Safety

Two severe toxicities were reported: diarrhea (cohort C; also reported as a grade 3 SAEs) and decreased left ventricular ejection fraction (cohort B; also reported as a grade 2 AE).

Grade 3 AEs considered by the investigator to be related/possibly related to WT1-immunotherapeutic administration were reported by one patient in cohort A (headache, two separate events) and one patient in cohort C (diarrhea); the latter was also reported as a SAE and as a severe toxicity event (Table 1).

**Table 1:** Overall incidence of AEs and SAEs (total treated cohort)

	Cohort A (*N* = 22)		Cohort B (*N* = 15)		Cohort C (*N* = 15)		Cohort D^a^ (*N* = 8)
	WT1 (*N* = 15) *n* (%)	Placebo (*N* = 7) *n* (%)	WT1 (*N* = 9) *n* (%)	Placebo (*N* = 6) *n* (%)	WT1 (*N* = 11) *n* (%)	Placebo (*N* = 4) *n* (%)	WT1 *n* (%)
AEs							
Any	15 (100)	5 (71)	9 (100)	6 (100)	11 (100)	4 (100)	7 (88)
Grade 3–5^b^	3 (20)	0 (0)	3 (33)	6 (100)	7 (64)	1 (25)	5 (63)
Related/possibly related^c^	12 (80)	2 (29)	3 (33)	0 (0)	7 (64)	1 (25)	6 (75)
Grade 3 related/possibly related^d^	1 (7)	0 (0)	0 (0)	0 (0)	1 (9)	0 (0)	0 (0)
SAEs	3 (20)	0 (0)	4 (44)	2 (33)	5 (45)	1 (25)	5 (63)

^a^Patients in cohort D received WT1-immunotherapeutic in an open-label manner

^b^AEs of grade 3 or higher intensity

^c^AEs considered by the investigator to be related or possibly related to WT1-immunotherapeutic/placebo administration

^d^AEs of grade 3 intensity considered by the investigator to be related or possibly related to WT1-immunotherapeutic/placebo administration

*WT1* WT1-immunotherapeutic; *AEs* adverse events; *SAEs* serious adverse events; *N*, number of patients; *n* (%), number (percentage) of patients reporting at least once the AE

Thirty-seven SAEs were reported by 20 patients (Table 1); two were considered by the investigators to be related/possibly related to WT1-immunotherapeutic administration: grade 2 polymyalgia rheumatica (cohort A; also reported as potential immune-mediated disorder) and diarrhea (mentioned above).

Two patients (WT1 group, cohort B) died during the study. One patient died due to an unknown cause, possibly due to underlying medical conditions of hypertension and thrombosis; this fatal SAE was assessed by the investigators as not causally related to WT1-immunotherapeutic administration. The second patient died due to progressive BC.

The Data Safety Monitoring Committee reviewed safety data every six months during the trial, with the last review in June 2015, and did not identify any potential safety issues.

### Immunogenicity

At baseline, all patients were seronegative for WT1-specific antibodies; post-dose 4, all 10 patients from cohort A (100%), 0/8 patients (0.0%) from cohort B, 6/11 (54.5%) patients from cohort C, and 2/3 (66.7%) patients from cohort D were humoral responders.

**Fig. 2:**
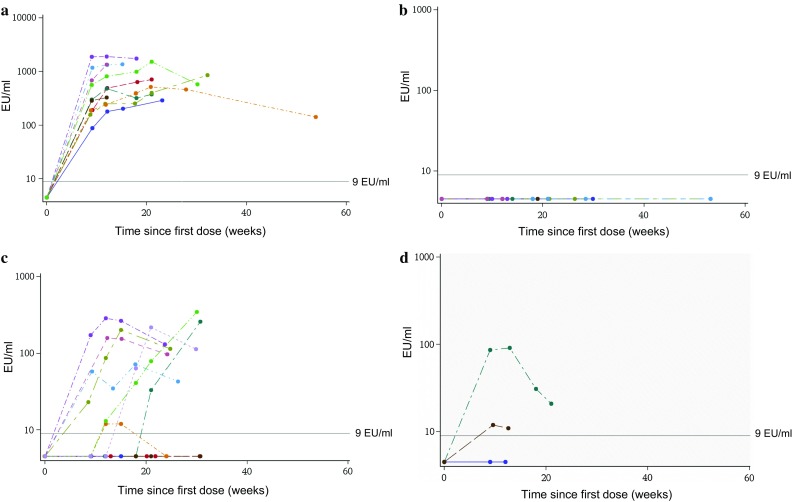
Pre- and post-immunization WT1-specific antibody titers in patients from **a** cohort A, **b** cohort B, **c** cohort C, and **d** cohort D (ATP cohort for immunogenicity). *ATP* according-to-protocol; EU/ml, ELISA units per ml (antibody concentration). The cut-off of the ELISA assay was 9 EU/ml. The color lines correspond to individual patients’ antibody titers at indicated timepoints

The highest WT1-specific antibody levels were observed in cohort A, in which patients received AIs as concomitant standard treatment (Fig. 2a). No antibody response was observed in cohort B receiving concomitant chemotherapy (Fig. 2b), while in cohorts C and D, weak WT1-specific antibody responses were only observed in some patients (Fig. 2c–d).

Of note, different types of antibody responses were observed in cohort C, with some patients presenting no antibody response (similar to cohort B), some having a delayed response, and others, immediate antibody titer development. WT1-specific antibody titers of patients from cohort C who developed an immune response were around 1 log below the results obtained in cohort A. Patients from cohort C who were immediate antibody responders received docetaxel, carboplatin, and trastuzumab (TCH) as concomitant chemotherapy. In cohort B, nearly all patients received sequential chemotherapy, starting with the combination of anthracyclines/cyclophosphamide and finishing with taxane-based therapy (paclitaxel or docetaxel). Patients in cohort C with no or a delayed immune response received the same treatment combination as in cohort B with the addition of trastuzumab.

No conclusions could be drawn for cohort D, as antibody responses were evaluated for only 3/8 patients enrolled in TTC due to the early termination of the study; two of these patients showed positive responses within the same range as those observed in cohort C.

### Clinical activity

The clinical activity was evaluated in 51 patients and is shown by treatment group in Table 2. In cohort A, among the 18 evaluable patients, seven patients had PR and 11 had no response. Among 15 patients in cohort B, two had pCR, eight had PR, and five patients had no response. Of the ten evaluable patients in cohort C, nine had pCR, four had PR, and one patient had no response. Among the four patients from cohort D who received WT1-immunotherapeutic in an open manner, one had pCR and three had PR.

**Table 2:** Overall pathological response rates (total treated cohort)

0		No response		Partial response		pCR	pCR rate^a^
Cohort	Group	Grade 1 n (%)	Grade 2 n (%)	Grade 3 n (%)	Grade 4 n (%)	Grade 5 n (%)	
A (*N* = 19)	WT1 (*N* = 13)	6 (50.0)	2 (16.7)	4 (33.3)	0 (0.0)	0 (0.0)	3–5%
		8 (66.7)		4 (33.3)			
	Placebo (*N* = 6)	2 (33.3)	1 (16.7)	3 (50.0)	0 (0.0)	0 (0.0)	
		3 (50.0)		3 (50.0)			
B (*N* = 15)	WT1 (*N* = 9)	4 (44.4)	0 (0.0)	4 (44.4)	1 (11.1)	0 (0.0)	6–30%
		4 (44.4)		5 (55.6)			
	Placebo (*N* = 6)	0 (0.0)	1 (16.7)	3 (50.0)	0 (0.0)	2 (33.3)	
		1 (16.7)		3 (50.0)			
C (*N* = 15)	WT1 (*N* = 11)	0 (0.0)	1 (10.0)	1 (10.0)	2 (20.0)	6 (60.0)	30–65%
		1 (10.0)		3 (30.0)			
	Placebo (*N* = 4)	0 (0.0)	0 (0.0)	1 (25.0)	0 (0.0)	3 (75.0)	
		0 (0.0)		1 (25.0)			
D (*N* = 4)^b^	WT1	0 (0.0)	0 (0.0)	2 (50.0)	1 (25.0)	1 (25.0)	
		0 (0.0)		3 (75.0)			

^a^pCR rate: pCR rate under standard treatment for a given patient population reported in literature

^b^All patients in cohort D received WT1-immunotherapeutic in an open-label manner

*N* number of patients; *pCR* pathological complete response; *n* number of patients in a given category; %, n/number of patients with available results × 100

## Discussion

The role of the host immune response to the tumor in BC has long been debated as, compared to melanoma or renal cell carcinoma, BC has been considered less immunogenic. However, current data suggest that BC, particularly the more aggressive subtypes of HER2-positive and triple-negative BC, can elicit host antitumor immune responses, and that the robustness of the response correlates with prognosis [5, 19–21]. The concept of natural immunogenicity of BC is based on the presence of tumor-infiltrating lymphocytes (TILs) and other immune cells within the tumor microenvironment, on the prognostic value of immune-related gene signatures, and the frequency of genetic instability which leads to higher numbers of somatic mutations and neoantigens [5, 22]. Additionally, the pre-existing immunologic response might enhance the effects of conventional chemotherapy [5, 23].

In cohort A, all patients who received WT1-immunotherapeutic developed WT1-specific antibodies. The antibody titers obtained in this cohort can be considered as reference titers, as only in this cohort patients did not receive chemotherapy or routine corticosteroids. In contrast, none of the patients receiving WT1-immunotherapeutic in cohort B developed antibodies. Analysis of B-cell population dynamics revealed depletion of B-cells in these patients compared to healthy donors, either due to the chemotherapy itself or the corticosteroids which are routinely used as anti-emetics in patients receiving chemotherapy (data not shown). The impact of cancer treatments on all lymphocytic populations, especially B-cells, has been previously described [24–26]. A study in BC patients evaluating the effects of combination chemotherapy regimens with epirubicin (5-fluorouracil, epirubicin, cyclophosphamide) versus doxorubicin (5-fluorouracil, doxorubicin, cyclophosphamide) on immune cells, revealed an increase in cytotoxic T-cell levels and natural killer cell levels, and a dramatic decrease in B-cell levels in the blood following in either regimen [26]. Nevertheless, the lympho-depleting effects induced by chemotherapy are transient and soon after drug discontinuation, a homeostatic rebound overshoot of the lymphocytic pool occurs [24].

In cohort C, a mix of titer profiles was observed, supporting the hypothetic blunting effect of chemotherapy co-administered on day 1, and also suggesting that different chemotherapy agents may have differing immunosuppressive effects. Diverse myelosuppressive effects of specific chemotherapeutic agents have been previously reported [27–29].

Another parameter difficult to discriminate from the chemotherapy effect is the impact of co-administered corticosteroids which were allowed per protocol for the prevention and treatment of chemotherapy-related nausea and hypersensitivity reactions. In cohort C, patients received trastuzumab co-administered with chemotherapy, and in numerous cases, patients receiving chemotherapy also received corticosteroids.

The traditional paradigm that chemotherapeutic agents suppress immune response has been challenged by evidence that chemotherapy induces, and is dependent upon activation of certain immunologic effects and may promote immune-mediated tumor destruction [30–33]. TILs within breast tumors have been shown to correlate with pCR and clinical response to neoadjuvant chemotherapy [34, 35]. The possible immunomodulatory mechanisms involving trastuzumab include inhibition of HER2-mediated signaling and antibody-dependent cell-mediated cytotoxicity [36, 37]. The AI anastrozole was shown to alter the proinflammatory cytokine levels and suppressed differentiation of naive T-cells into regulatory T-cells, which are known to produce immunosuppressive cytokines in the tumor microenvironment [38, 39].

An additional cohort D received WT1-immunotherapeutic on day 14 of each chemotherapy cycle, to evaluate if delaying the immunotherapy administration after the chemotherapy treatment improves the immune response. Day 14 was selected because corticosteroids were not administered on that day and patients were expected to have passed their white cell count nadir. In a study with MAGE-A3 immunotherapeutic in non-small cell lung cancer patients who received concurrent cisplatin/vinorelbine chemotherapy regimen, a robust MAGE-A3-specific antibody response was induced in all patients [40]. However, in this previous study, MAGE-A3 immunotherapeutic was administered on day 8 of each chemotherapy cycle, whereas in cohort B of the current study, chemotherapy was administered on the same day as WT1-immunotherapeutic. This information also reinforced the hypothesis of a differential impact of the chemotherapy types on the immune response. Although our study was stopped before finalization of enrollment in cohort D, from the few data collected, it is apparent that delaying administration of immunotherapy (14 days following the chemotherapy cycle initiation) did not improve the immunogenicity, as antibody titers obtained in cohort D were similar to those obtained in cohort C. In one patient from cohort D, the sequence of chemotherapy was reversed, starting with docetaxel followed by epirubicin/cyclophosphamide combination. In this patient, the WT1-specific antibody level rose immediately while the patient underwent docetaxel chemotherapy, but fell thereafter following epirubicin/cyclophosphamide treatment. Altogether, these data suggest that concomitant corticosteroid administration and/or possibly specific chemotherapies (particularly anthracycline combinations) impacted the WT1-specific antibody generation post-vaccination.

Limitations of our study include the presence of multiple confounding factors and small numbers of patients in each cohort.

In conclusion, concurrent administration of WT1-immunotherapeutic and standard therapy was well tolerated and induced WT1-specific antibody response in BC patients when co-administered with neoadjuvant AIs. In patients on neoadjuvant chemotherapy or a trastuzumab–chemotherapy combination, the humoral response was impaired or blunted, likely due to either co-administration of corticosteroids and/or the chemotherapies themselves.

## Electronic supplementary material

Below is the link to the electronic supplementary material.

Supplementary material 1 (DOCX 834 kb)
