# Supplementary figures and images for: Safety and immunogenicity of neoadjuvant treatment using WT1-immunotherapeutic in combination with standard therapy in patients with WT1-positive Stage II/III breast cancer: a randomized Phase I study
Source: Breast Cancer Res Treat. 2017 Feb 7;162(3):479–88. doi: 10.1007/s10549-017-4130-y (PMC5332485; doi:10.1007/s10549-017-4130-y)

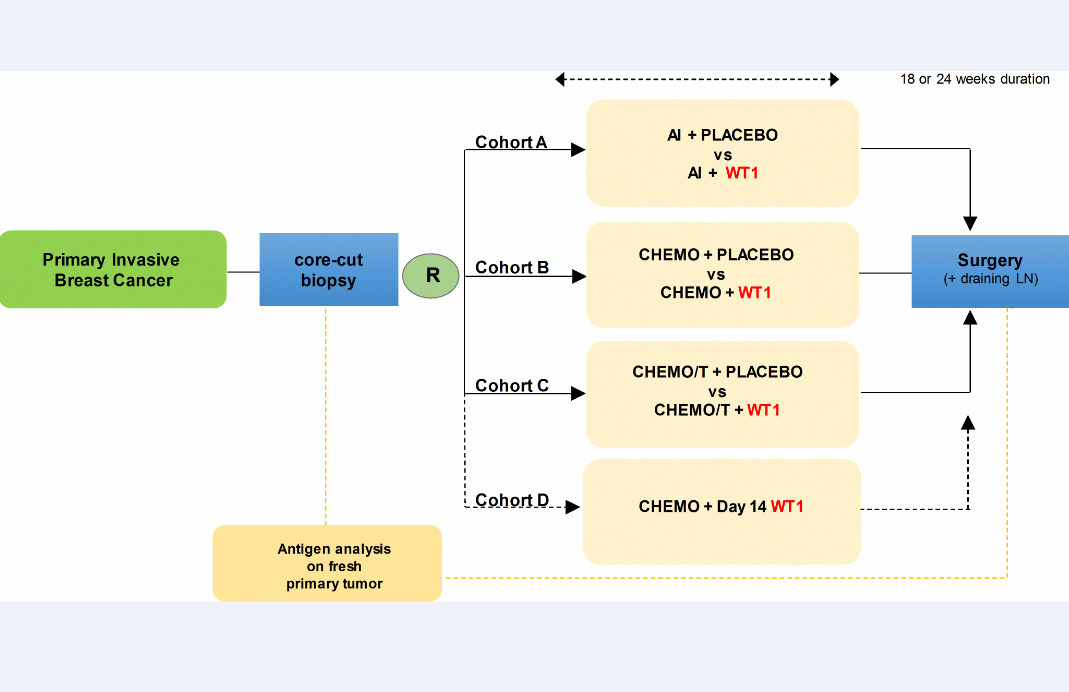


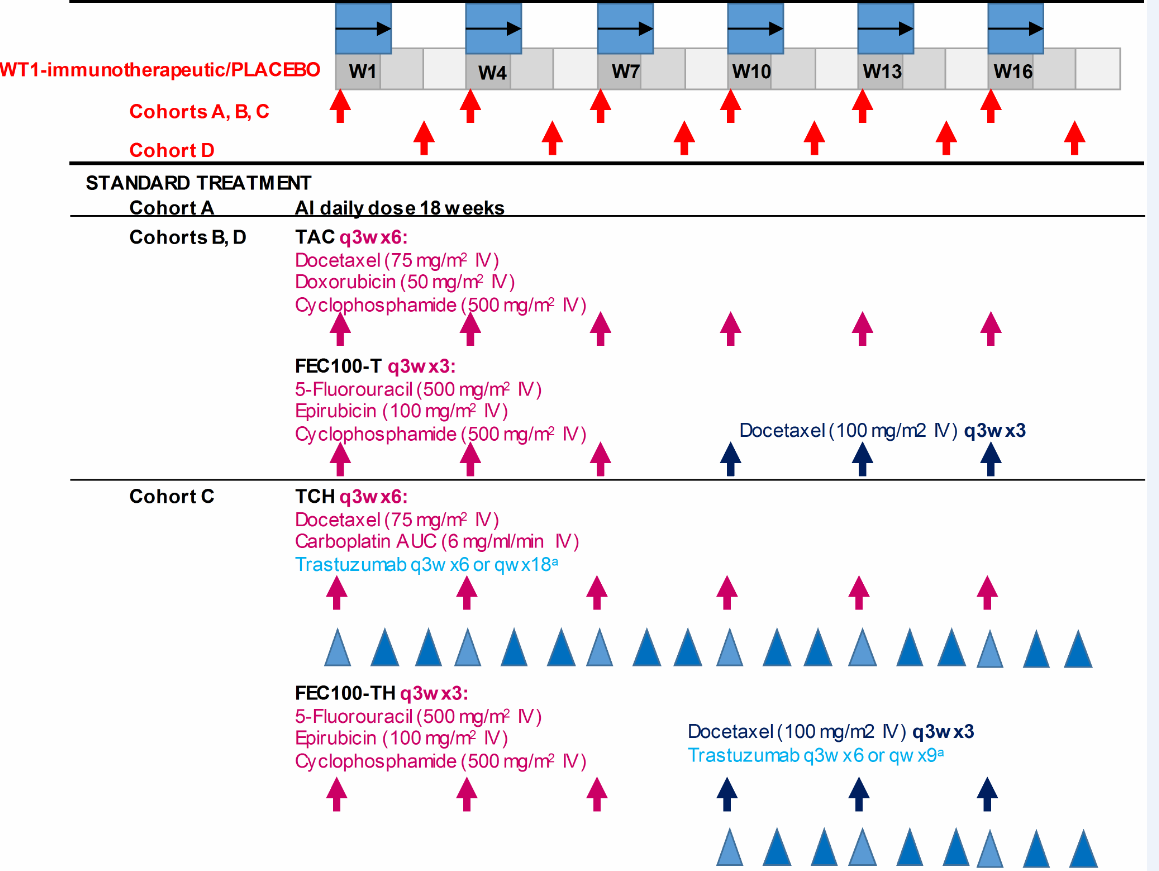


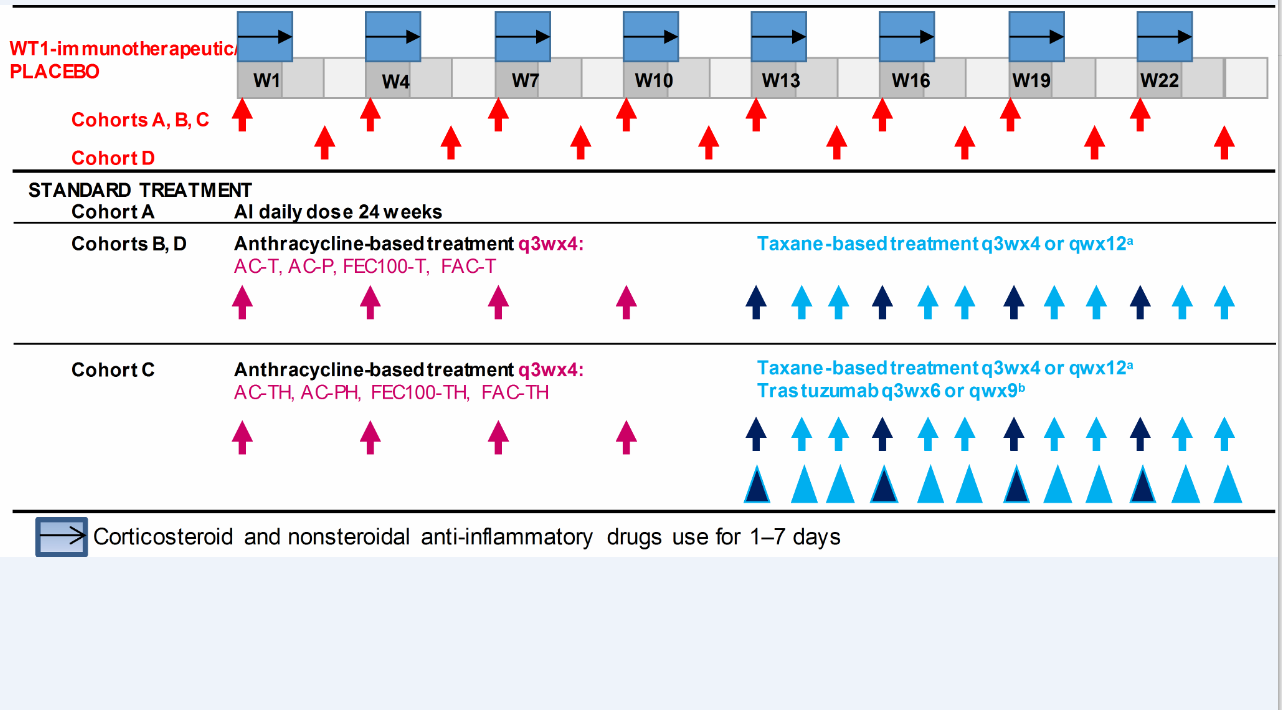


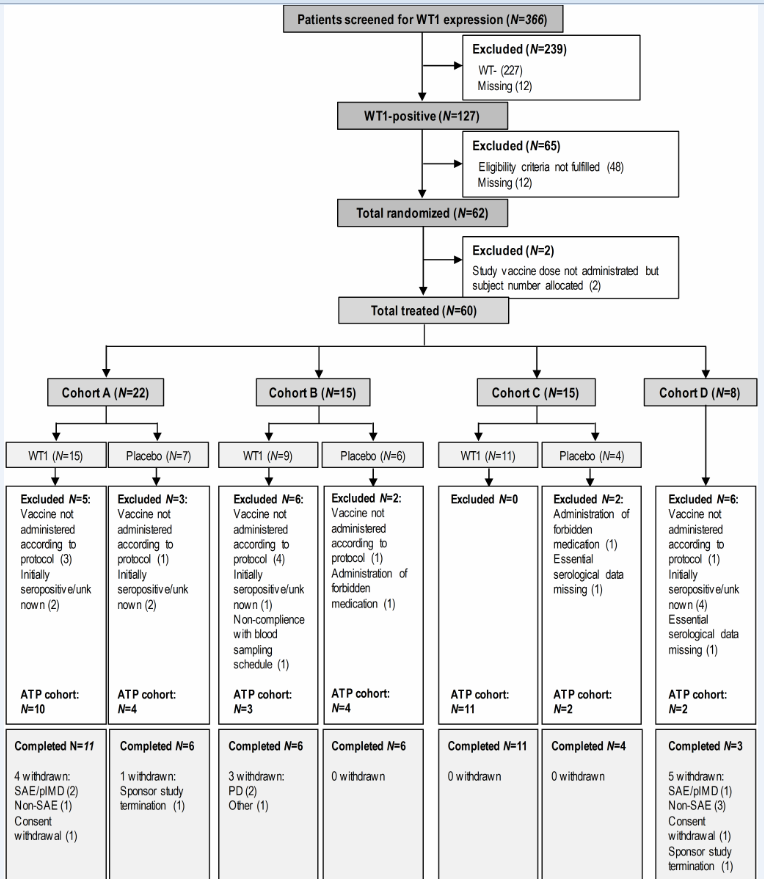

Supplement: Supplementary file 1 — Supplementary material 1 (DOCX 834 kb) [file 10549_2017_4130_MOESM1_ESM.docx]
